# Esophageal lung misdiagnosed as tracheoesophageal fistula: A case report

**DOI:** 10.1016/j.radcr.2022.12.029

**Published:** 2023-01-12

**Authors:** Elham Zarei, Nima Rakhshankhah, Nasrin Hoseiny Nejad, Alireza Eshghi

**Affiliations:** a Assistant Professor of Radiology, Ali Asghar Children Hospital, Iran University of Medical Sciences (IUMS), Tehran, Iran; b Department of Radiology, Iran University of Medical Sciences (IUMS), Hemat Highway next to Milad Tower, 14535, Tehran, Iran; c Department of Pediatric Pulmonology, Ali Asghar Children Hospital, School of Medicine, Iran University of Medical Sciences, Tehran, Iran

**Keywords:** Esophageal atresia, Tracheoesophageal fistula, Bronchopulmonary dysplasia, Infant, Esophageal lung, NICU, neonatal intensive care unit, PICU, pediatric intensive care unit, IVF, in vitro fertilization, CXR, chest X-ray, CBPFM, communicating bronchopulmonary foregut malformations, TEF, tracheoesophageal fistula

## Abstract

An esophageal lung is a subtype of communicating bronchopulmonary foregut malformation (CBPFM) where the lung, often right side, communicates with the esophagus, which causes hypoplastic and consolidated diseased lung, and is usually diagnosed late in its clinical course. Clinical suspicion based on patient history, signs, and symptoms should lead to this opinion. A chest CT scan combined with esophagography is highly recommended for suspicious cases. Here, we describe the case of a 3-month-old female infant who was referred to our hospital because of respiratory distress. The diagnosis of the congenital esophageal lung was made following a chest CT scan and esophagography. This is a very rare case that is misdiagnosed as a tracheoesophageal fistula. CBPFMs are rare abnormalities caused by an abnormal connection between the respiratory tract and the gastroesophageal system. Early diagnosis and differentiation of these abnormalities from sequestration and tracheoesophageal fistula could improve the outcome.

## Background

Communicating bronchopulmonary foregut malformations (CBPFMs) are rare developmental abnormalities specified by a connection between the respiratory tract and the gastroesophageal system [1,2]. An esophageal lung is a subtype of CBPFM where the lung, often right side, connects with the esophagus, which causes a hypoplastic and consolidated, diseased lung [3]. This article reports a patient with a congenital esophageal lung that was misdiagnosed as a tracheoesophageal fistula. Our objective is to establish information that can help doctors find these patients as soon as possible, so that patient outcomes would improve.

## Case presentation

A 3-month-old female was referred to our hospital because of a fever associated with respiratory distress. She was a result of a 38-week pregnancy started by in vitro fertilization (IVF).

In the first week of life, she had been hospitalized in another hospital's neonatal intensive care unit (NICU) due to respiratory distress following feeding. At that time the patient had been discharged suspicious of pulmonary hypoplasia regarding its chest X-ray (CXR) findings. She had mild tachypnea since then without readmission.

**Fig. 1:**
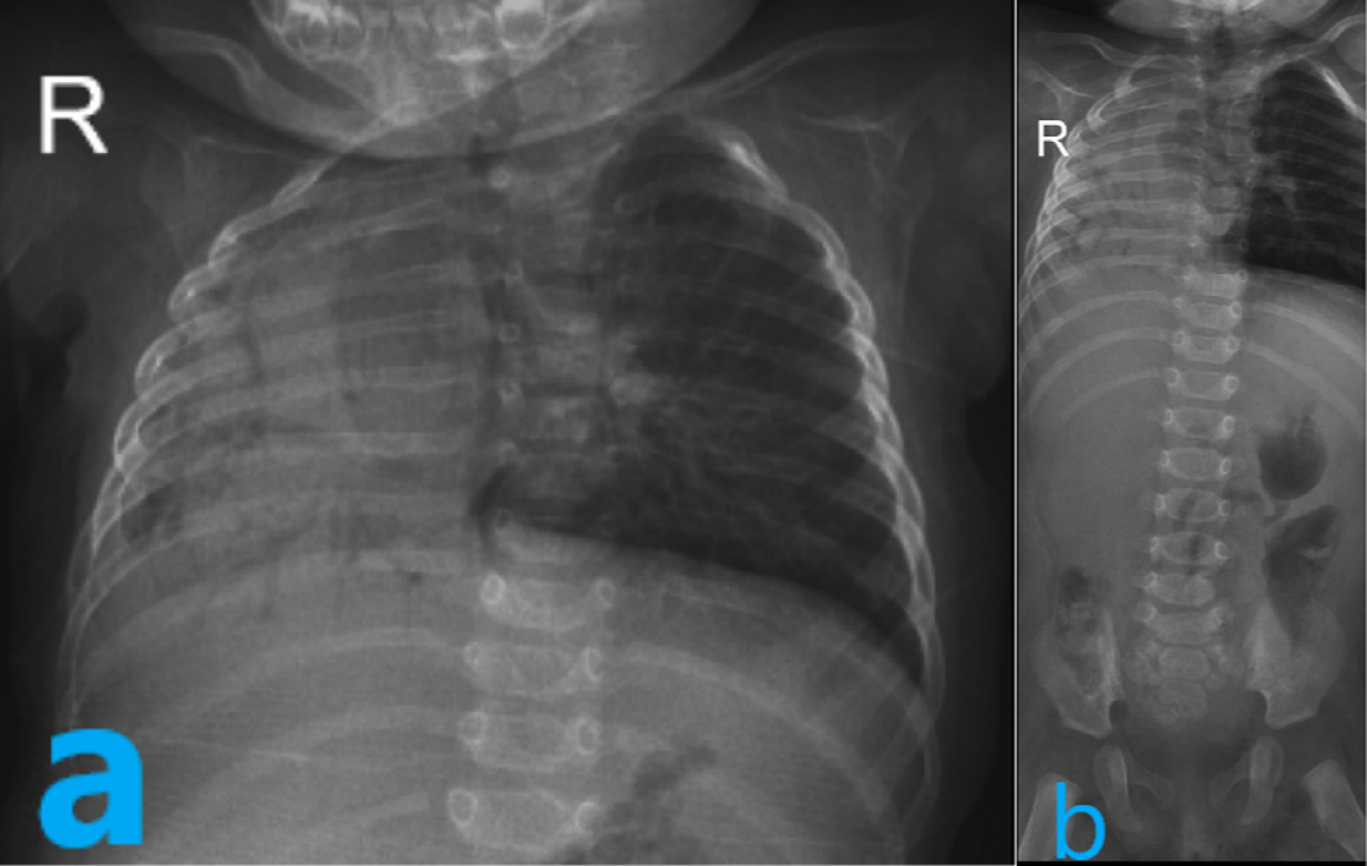
Chest X-ray (a) shows the atelectatic right lung contained air bronchograms with the abnormal position of the right main bronchus approximately at the base of the thorax. Evidence of hypoplasia of the sacrum was also observed in the thoracoabdominal X-ray (b).

In the third month of her life, she was referred to our hospital while she was febrile and her tachypnea worsened. Her O2 saturation without supplementary oxygen was 85%. On auscultation, the right lung was silent, and the heart sound was heard in the right sternal border. CXR showed near to complete opacification of the right lung with mediastinal shift to the right side and compensatory hyperaeration of the left lung. The atelectatic right lung contained air bronchograms with the abnormal position of the right main bronchus approximately at the base of the thorax (Fig. 1a). In addition, evidence of hypoplasia of the sacrum was also observed in the thoracoabdominal X-ray (Fig. 1b).

Abdominal sonography and echocardiography were done and didn't show remarkable findings.

**Fig. 2:**
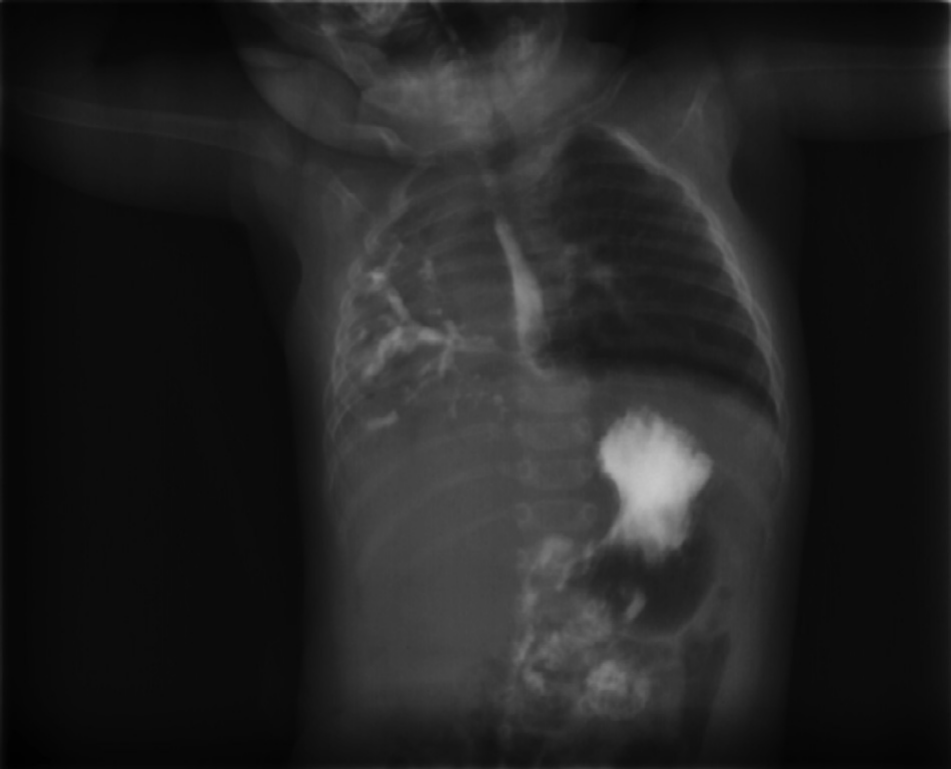
Water-soluble esophagography shows the anomalous origin of the right main bronchus from the distal part of the esophagus. No evidence of tracheoesophageal fistula is seen.

A diagnosis of lung hypoplasia was made for the infant, and according to the history of respiratory symptoms following feeding, with the suspicion of fistula, an esophagography with water-soluble contrast material was requested for the patient (Fig. 2).

**Fig. 3:**
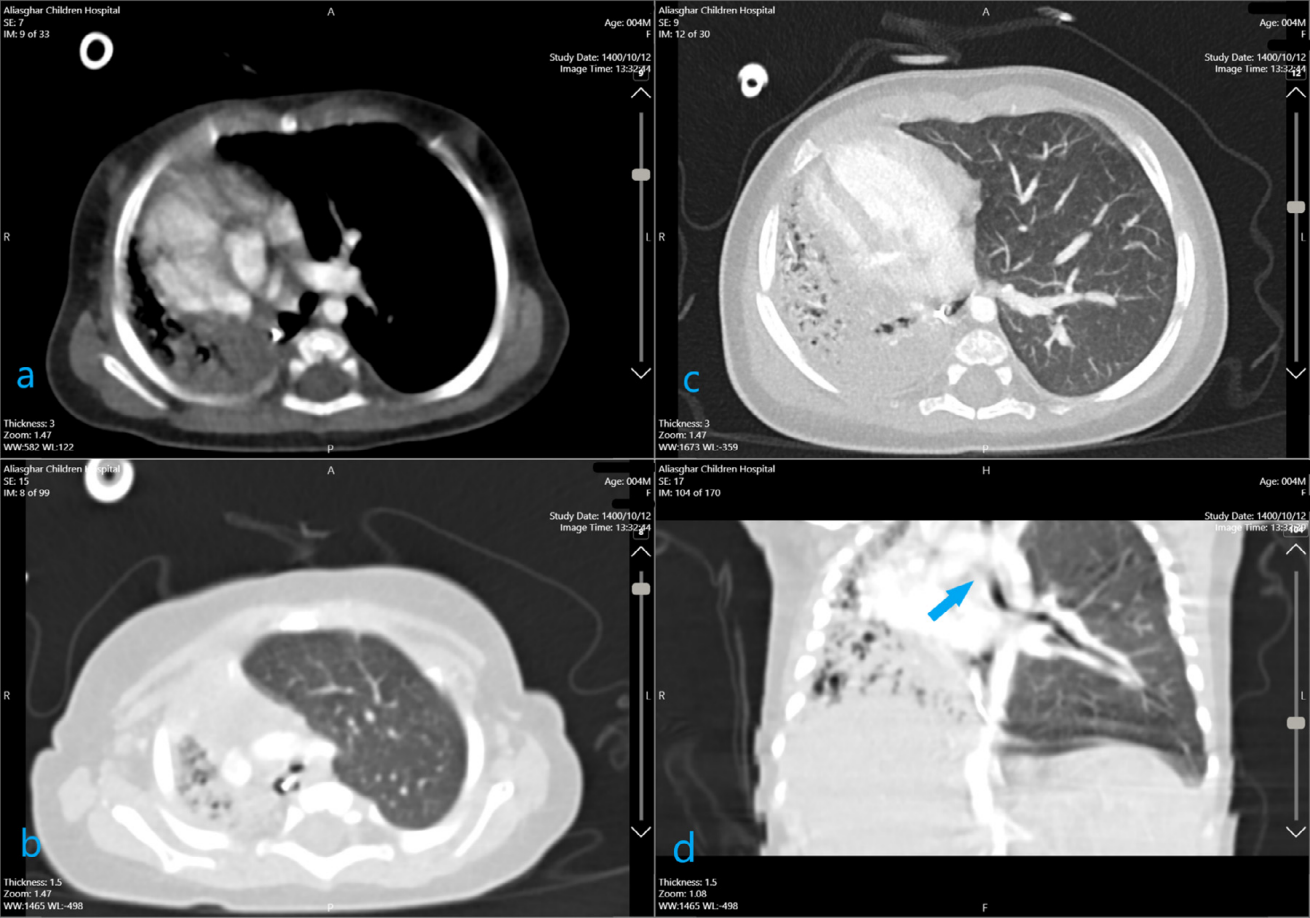
IV contrast chest CT scan shows a hypoplastic right lung with hypoplastic normal origin pulmonary vessels (a). No clear connection is seen between the esophagus and the right main bronchus (b, c) but the right bronchus was absent at the level of the carina (arrow in d) and is seen almost at the base of the right hemi thorax on the right side of the esophagus.

With the diagnosis of lung hypoplasia along with esophageal lung on the right side, a chest CT scan with IV contrast was performed and showed a hypoplastic right lung with hypoplastic normal origin pulmonary vessels. No clear connection was seen in the CT scan between the esophagus and right main bronchus but the right bronchus was absent at the level of the carina and was seen almost at the base of the right hemi thorax on the right side of the esophagus (Fig. 3).

**Fig. 4:**
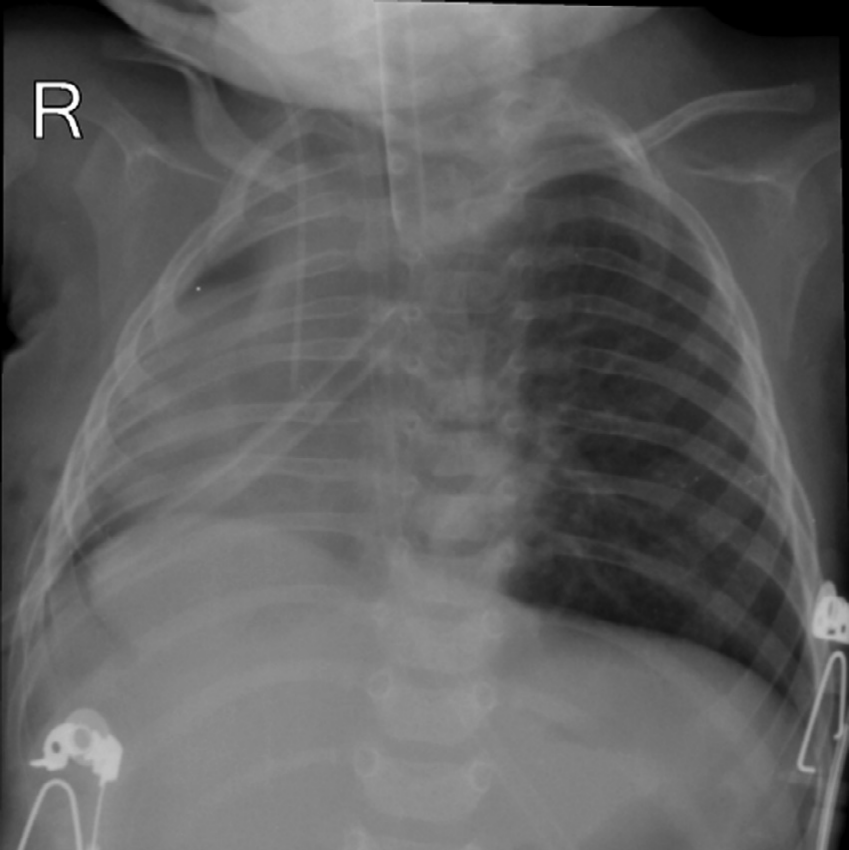
Postoperation chest X-ray shows right pneumonectomy with partial pneumothorax and left side hyperinflation. Also intubation tube and central venous catheter is seen in appropriate positions.

After diagnosis the patient underwent surgery. Right thoracotomy was performed with posterolateral incision. The right lung was hypoplastic and the bronchus of the right lobe originated from the distal thoracic esophagus. After ligation, the right lung could not be expanded and a pneumonectomy was performed. After the operation, the patient was admitted to the pediatric intensive care unit (PICU) (Fig. 4). On the second day after the operation, she was extubated, but her ventilation was insufficient and she expired due to difficult intubation and respiratory insufficiency.

## Discussion

The trachea and esophagus originate from a similar primitive foregut. These tracts are separated by epithelial grooves at 4 weeks of gestation. The foregut divides into a collageomembranous tube in the ventral, which is composed of incomplete cartilaginous rings in an anterior arc and membranous wall posteriorly, and a digestive tube in the dorsal [4]. Division defects such as atresias, webs, and esophageal lung or bronchus result from foregut developmental abnormalities in the esophagus and trachea. The abnormal tract between the esophagus and lung probably originated from an embryonic lung bud that fails normal branching and abnormalities such as web is probably caused by incomplete recanalization [4,5]. The clinical importance of these lesions is in those patients requiring hospitalization as they are more susceptible to recurrent pulmonary infections and respiratory failure [6]. Suspicion is usually made within the first year of life when the infant shows recurrent pulmonary infections, respiratory failure, and failure to thrive [7].

CBPFMs are recently classified into 5 groups as follows [7,8]:

1A: Total separated lung connecting with the gastroesophageal tract, associated with TEF and esophageal atresia

1B: separated anatomic lobe or segment connecting with the gastroesophageal tract, associated with TEF and esophageal atresia

II: Total separated lung connecting with the lower esophagus; absent ipsilateral main stem bronchus

III: Isolated anatomic lobe or segment connecting with the gastroesophageal tract

IV: Portion of the normal bronchial system connecting with the esophagus

Our case was classified as category II where the entire lung was aerated by the lower esophagus without ipsilateral main stem bronchus. As we saw in our case, because of the proximity of the right bronchus to the esophagus, the right lung and bronchus are frequently affected [9].

Diagnosis is usually suspected by CXR and CT scan which shows bronchiectasis, hypoplastic consolidated lung tissue in the region aerated by the esophageal lung [1]. Esophagography together with bronchial and esophageal endoscopy are modalities that confirm the diagnosis [9,10].

## Conclusions

CBPFMs are rare abnormalities caused by an abnormal connection between the respiratory tract and the gastroesophageal system. In lung hypoplasia or stable one-sided lung collapse in children, especially if they have symptoms of frequent respiratory infection after feeding, it is important to evaluate the origin of the airway to differentiate CBPFMs from sequestration and tracheoesophageal fistulas.

In addition, if there are air bronchograms in the hypoplastic lung in the CXR, its path should be taken care of.

## Authors’ contributions

All authors contributed to the study's conception and design. The first draft of the manuscript was written by NR and EZ, NHN and AE commented on previous versions of the manuscript. All authors read and approved the final manuscript.

## Availability of data and materials

All data generated or analyzed during this study are included in this article and are available at Ali Asghar Clinical Research center (AACRDC), Iran University of Medical Sciences (IUMS), Tehran, Iran.

## Ethics approval

This manuscript is a human case report.

## Patient consent

I declare that the patient's guardian is fully informed about the research and give me permission in full consciousness to use of photographs, clinical and laboratory data of patient. Written informed consent is taken from patient's guardian.
